# Maintenance of stem cell activity in plant development and stress responses

**DOI:** 10.3389/fpls.2023.1302046

**Published:** 2023-12-12

**Authors:** Huankai Zhang, Yangwei Mu, Hui Zhang, Caiyu Yu

**Affiliations:** ^1^ College of Life Sciences, Zaozhuang University, Zaozhuang, China; ^2^ National Key Laboratory of Wheat and Maize Crop Science, College of Life Sciences, Henan Agricultural University, Zhengzhou, China

**Keywords:** stem cell, meristem, DNA damage, ROS, Ca^2+^, abiotic stress

## Abstract

Stem cells residing in plant apical meristems play an important role during postembryonic development. These stem cells are the wellspring from which tissues and organs of the plant emerge. The shoot apical meristem (SAM) governs the aboveground portions of a plant, while the root apical meristem (RAM) orchestrates the subterranean root system. In their sessile existence, plants are inextricably bound to their environment and must adapt to various abiotic stresses, including osmotic stress, drought, temperature fluctuations, salinity, ultraviolet radiation, and exposure to heavy metal ions. These environmental challenges exert profound effects on stem cells, potentially causing severe DNA damage and disrupting the equilibrium of reactive oxygen species (ROS) and Ca^2+^ signaling in these vital cells, jeopardizing their integrity and survival. In response to these challenges, plants have evolved mechanisms to ensure the preservation, restoration, and adaptation of the meristematic stem cell niche. This enduring response allows plants to thrive in their habitats over extended periods. Here, we presented a comprehensive overview of the cellular and molecular intricacies surrounding the initiation and maintenance of the meristematic stem cell niche. We also delved into the mechanisms employed by stem cells to withstand and respond to abiotic stressors.

## 1 Introduction

Pluripotent stem cells, the versatile architects of multicellular organisms, orchestrate the continual regeneration of organs and tissues, mending the wear and tear of daily life. This remarkable regenerative potential, harnessed by some plants, enables them to persist for centuries or even millennia (Sang et al., 2018; McKim, 2019; Hata and Kyozuka, 2021; Umeda et al., 2021). Stem cells, nestled within their specialized microenvironments, are key to orchestrating cellular differentiation. These cells, initially undifferentiated, retain the remarkable ability for self-renewal and the generation of progeny. These progenies, in turn, may either retain stem cell properties or embark on a journey towards specialized specific functions (Aichinger et al., 2012; Wang et al., 2020; Nicolas and Laufs, 2022). However, continuous differentiation ultimately curtails the ability of cells to sustain continuous proliferation, underscoring the critical importance of precise stem cell regulation. Any deviation, whether in the form of a reduction or excessive proliferation of stem cells, can lead to pathological states, disrupting the cellular composition and impairing tissue function. Recent scientific endeavors in stem cell research have been predominantly devoted to unraveling the intricacies of initiation, maintenance, and signal transduction (Sablowski, 2011; Janocha and Lohmann, 2018; Warghat et al., 2018; Motte et al., 2019; Fujinami et al., 2020; Xue et al., 2020), often involving the participation of plant hormones (Lee et al., 2013; Xu et al., 2020; Gomes and Scortecci, 2021; Yamoune et al., 2021; Li et al., 2023).

For sessile organisms like plants, the challenge of survival hinges on their ability to adapt to a repertoire of abiotic stresses, including drought, salinity, temperature fluctuations, exposure to heavy metal ions, ultraviolet radiation, and other various physical perturbations (Sun et al., 2020; Markham and Greenham, 2021; Zhang et al., 2022; Kopecka et al., 2023). These stressors not only affect the geographical distribution of plants but also exert profound effects on their growth, development, and, in agricultural contexts, crop yields (Villalobos-Lopez et al., 2022). The molecular landscape of plant responses to abiotic stresses is intricate, including signal perception, signal transduction, transcriptional regulation, post-transcriptional processing, translation, and post-translational modifications. Given the pivotal role of stem cells in plant development, contemporary research has increasingly focused on elucidating the relationship between plant stem cells and their responses to abiotic stresses (Gong et al., 2020; Lamers et al., 2020; Li et al., 2021a; Ubogoeva et al., 2021; Shinozaki and Yamaguchi-Shinozaki, 2022; Charng et al., 2023). In this review, we summarized the knowledge surrounding the composition, initiation, and maintenance of plant stem cells, as well as the multifaceted mechanisms governing plant responses to abiotic stresses. Particular emphasis was placed on the intricacies of signal perception, with a primary focus on the relationship between plant stem cells and abiotic stresses.

## 2 Stem cells of the shoot apical meristem

In plants, the distribution of stem cells spans roots, stems, and vascular tissues, serving as the self-sustaining units for organ development. These stem cells, nestled within the apical meristem, play a pivotal role in the postembryonic development of plants, including the shoot apical meristem (SAM) responsible for aboveground growth and the root apical meristem (RAM) governing root system development. The meristems possess dual functions, orchestrating both the production of new cells and the initiation of organogenesis (Aichinger et al., 2012; Liu et al., 2018a).

**Figure 1:**
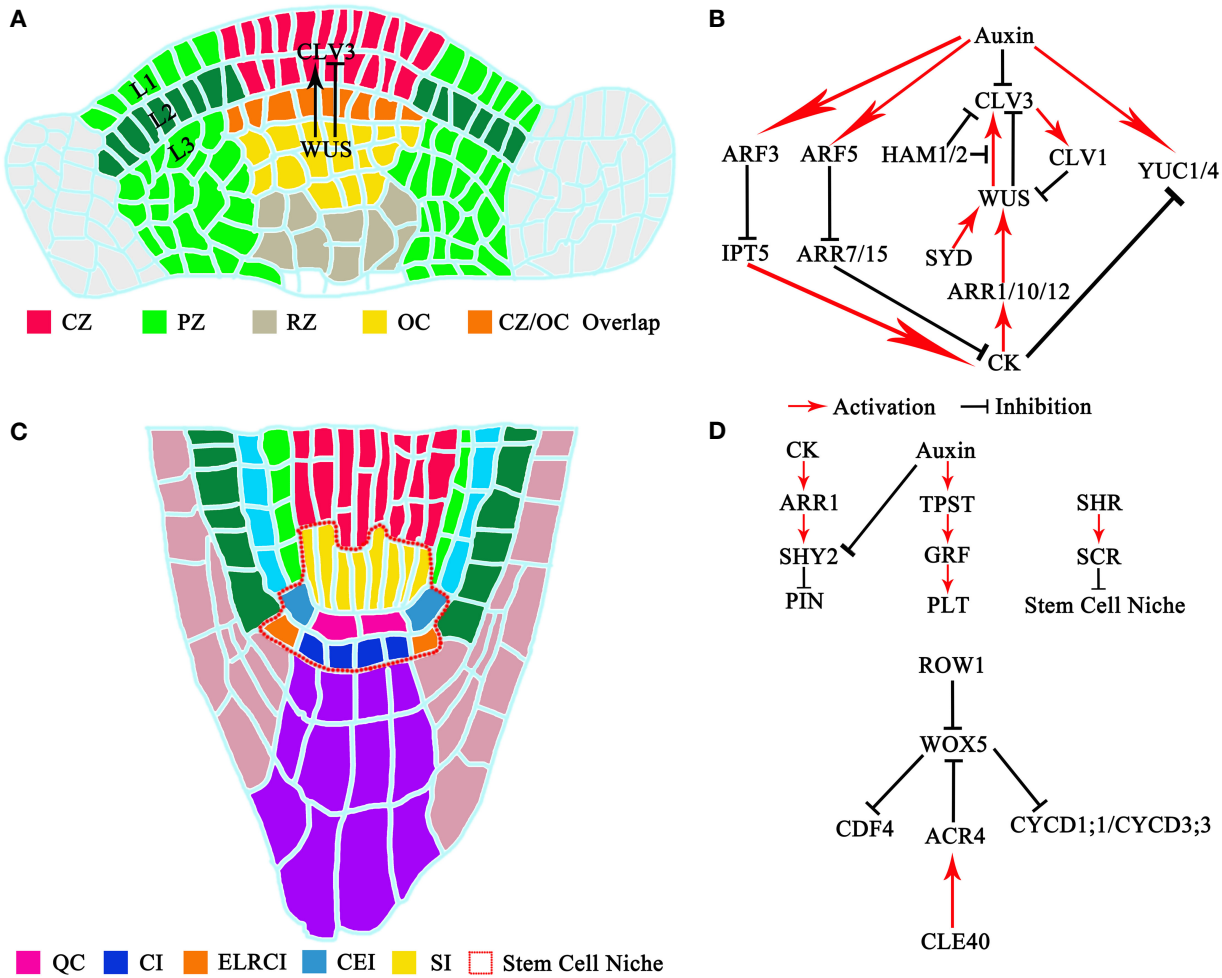
Structure and signaling pathways of meristem stem cells: **(A)** Structure of shoot meristem stem cells. CZ, central meristem zone; PZ, peripheral meristem zone; RZ, rib meristem zone; OC, organizer center. **(B)** Signaling pathways of shoot meristem stem cells. **(C)** Structure of root meristem stem cells. QC, quiescent center; CI, columella initial; ELRCI, epidermis or lateral root cap initial; CEI, cortex or endodermis initial; SI, stele initial. **(D)** Signaling pathways of root meristem stem cell.

The intricate mechanism of new cell generation within the shoot meristem has been illuminated through elegant cell-tracking experiments. Within the shoot meristem of *Arabidopsis thaliana* seedlings, cells in the SAM are distinctly organized into clonally separate layers. This arrangement, known as the tunica-corpus theory, classifies cells into the L1 and L2 layers forming the tunica and multiple layers at L3 and below constituting the corpus (Jurgens et al., 1994). This tri-layered structure characterizes Dicotyledons, while Monocotyledons possess two layers, and Gymnosperms feature just one. The vertical division of primordial cells residing in the L1 and L2 layers continuously expands the surface area of the plant shoot meristem (Wegner, 2000; Gross-Hardt and Laux, 2003). Further categorization of the SAM, according to the zoning theory, reveals the central meristem zone (CZ), rib meristem zone (RZ), and peripheral meristem zone (PZ) (Gross-Hardt and Laux, 2003). Stem cells reside at the apex of the CZ, with the cluster of cells beneath them, known as the organizer center (OC), playing a vital role in stem cell population maintenance (Kerstetter and Hake, 1997; Mayer et al., 1998; Lee and Clark, 2013; Tanaka et al., 2015; Somssich et al., 2016; Meng et al., 2017; Negin et al., 2017; Zhang et al., 2017; Su et al., 2020) (**Figure 1A**).

The initiation of the SAM involves key regulatory factors, *CLAVATA3* (*CLV3*) and *WUSCHEL* (*WUS*). The expression of the stem cell marker *CLV3* can be detected during embryonic development in the SAM. *WUS*, a pivotal regulator of stem cell homeostasis, is initially expressed in a 16-cell embryonic OC, dynamically maintaining the stem cell niche size (Mayer et al., 1998; Jha et al., 2020; Hirakawa, 2021). Plant hormones, especially auxin and cytokinin, assume a crucial role in regulating stem apical meristem initiation. *WOX2*, a *WUS* homologous gene, participates in apical meristem initiation by regulating PIN1-dependent auxin transport during embryogenesis (Lee et al., 2013; Zhang et al., 2017; Xu et al., 2020; Gomes and Scortecci, 2021; Yamoune et al., 2021; Li et al., 2023). In addition, various key regulatory factors contribute to stem cell initiation, such as *TOPLESS* (*TPL*), *ZWILLE* (*ZLL*), *SHOOTMERISTEMLESS* (*STM*), and *WOX2*. TOPLESS (TPL) encodes a member of the Groucho/Tup1-type transcriptional corepressors. TPL functions as a co-repressor for EAR-domain-containing transcription factors (Long et al., 2006; Szemenyei et al., 2008). *PLT* genes are direct targets of TPL. The phenotype of *tpl-1* in the shoot apex of mutant embryos is caused by ectopic expression of PLT genes (Smith and Long, 2010). Genetic interaction with *tpl-1* also casts the homeodomain-leucine zipper III (HD-ZIP III) transcription factors as key regulators of shoot fates (Smith and Long, 2010). HD-ZIP III proteins function in promoting “adaxial” fates in lateral organs and “central” fates in the meristem (Engstrom et al., 2004). Domain-specific expression of HD-ZIP III and PLT transcription factors establish shoot and root fates at the poles of the globular embryo (Jeong et al., 2011). Intriguingly, the *tpl-1* mutant phenotype manifests as the conversion of stems into roots, unveiling the pivotal role of the TPL protein. TPL engages in interactions with the auxin repressor IAA12/BDL, thereby mediating auxin-dependent transcriptional repression and influencing the initiation of shoot stem cells during embryogenesis (Long et al., 2006; Szemenyei et al., 2008). Another critical protagonist is the ZLL protein, whose expression is initially detected at the 2-cell and 8-cell embryonic stages. ZLL is one of the critical SAM regulators, which represses microRNA165/166 (miR165/166) for SAM maintenance. Levels of miR165/166 in the *zll* mutants are abnormally elevated, leading to a reduction in the quantity of *HD-ZIP III* gene transcripts, which is the target of miR165/166 (Liu et al., 2009). In *zll* mutants, elevated *ARF2* transcription levels and intensified auxin responses result in the absence of the shoot meristem. Therefore, ZLL initiates stem cell formation by negatively regulating the auxin signaling pathway (Moussian et al., 1998; Lynn et al., 1999; Tucker et al., 2008; Roodbarkelari et al., 2015). The formation of shoot stem cells also hinges on the STM protein. STM is involved in a negative feedback regulatory pathway with *CUP-SHAPED COTYLEDON* (*CUC*) and plays a pivotal role in initiating shoot stem cells (Takada et al., 2001; Hibara et al., 2006). This intricate relationship involves *CUC1* and *CUC2* inducing STM expression (Aida et al., 1999). Concurrently, as STM protein levels rise, they indirectly repress *CUC1* and *CUC2* gene expressions by activating miR-164a (Spinelli et al., 2011). Furthermore, *STM* induces stem cell initiation in a cytokinin-dependent manner (Jasinski et al., 2005; Yanai et al., 2005). The *WOX2* gene, in contrast, stimulates the up-regulation of the HD-ZIP III transcription factor, thereby regulating the expression of the cytokinin biosynthetic gene *ISOPENTENYLTRANSFERASE1* (*IPT1*), which is pivotal in the initiation of shoot stem cells (Zhang et al., 2017). It’s worth noting that ongoing research endeavors continue to unveil new key regulatory genes involved in shoot stem cell initiation, indicating the complexity of these processes. Plant hormones, particularly auxin and cytokinin, also exert significant influence during this stage.

The maintenance of shoot stem cells is governed by the *WUS/CLV* negative feedback pathway. Research has shown that the WUS protein relocates from the OC to the stem cell niche, thereby activating the expression of *CLV3* (Fletcher, 2018; Lopes et al., 2021), which in turn promotes stem cell production (Yadav et al., 2010; Yadav et al., 2011). Importantly, a dose-dependent relationship exists between the WUS protein levels and *CLV3* activation (Perales et al., 2016). In *Arabidopsis thaliana*, *CLV3* belongs to the CLV family, with *CLV1* and *CLV2* representing transmembrane protein kinases. A feedback regulatory pathway between *WUS* and *CLV3* ensues, where an increase in WUS protein expression promotes *CLV3* expression, consequently enhancing stem cell division. Conversely, escalating CLV3 protein secretion inhibits *WUS* expression, ensuring the maintenance of normal meristem development (Schoof et al., 2000; Song et al., 2010; Liu et al., 2013; Daum et al., 2014; Perales et al., 2016; Rodriguez et al., 2016; Su et al., 2020).

This negative feedback pathway also engages in interactions with factors in the cytokinin signaling pathway, particularly class B and class A Arabidopsis Response Regulator (ARR) transcription factors, collaboratively regulating the size of the SAM (Gordon et al., 2009; Hwang et al., 2012; Dai et al., 2017). Among these ARR proteins, class A ARRs are negative regulators of cytokinin, while class B ARRs are positive cytokinin regulators (Xie et al., 2018). The WUS protein, for instance, directly inhibits the expressions of several class A ARRs, such as ARR5, ARR6, ARR7, and ARR15 (Leibfried et al., 2005), consequently intensifying the cytokinin response in the SAM. In contrast, class B ARRs, including ARR1, ARR10, and ARR12, directly activate the *WUS* expression, contributing substantially to meristem homeostasis. Moreover, these ARRs directly repress the expression of *YUCs*, and indirectly promote WUS induction (Meng et al., 2017). This feedback regulation is critical for maintaining the SAM’s size and promoting overall stability (Chickarmane et al., 2012).

In addition to these feedback pathways, numerous other factors, frequently involving auxin or cytokinin signaling pathways, participate in the maintenance of the apical meristem. The classical auxin response model showed that intracellular auxin is transported by PIN family proteins in SAM and the auxin signal is sensed by auxin receptor *TRANSPORT INHIBITOR RESPONSE1 (TIR1)* (Vernoux et al., 2000; Kepinski and Leyser, 2005). Studies have illuminated that auxin can indirectly influence the *WUS-CLV3* feedback loop by inhibiting the expression of *ARR7/15* through MP/ARF5 (Zhao et al., 2010). The repressor ARF/ARF3, expressed in the peripheral SAM region, can directly impede the expression of the cytokinin synthesis gene *ISOPENTENYLTRANSFERASE5 (AtIPT5)*, thereby modulating auxin and cytokinin distribution in specific SAM regions. This distribution pattern, featuring auxin predominantly in the peripheral region and cytokinin chiefly in the central region, fosters stem cell activity and ensures the stable growth of the SAM (Cheng et al., 2013). Notably, the pivotal auxin response factor, MP/ARF5, an activator in the ARF, has been identified as a negative regulator of *CLV3* expression. This regulatory action operates through direct transcriptional inhibition of *DORNROSCHEN/ENHANCER OF SHOOT REGENERATION 1* (*DRN/ESR1*) (Luo et al., 2018). In addition, the collaborative synergy between the HAIRY MERISTEM (HAM1/2), proteins with GRAS domain which function in dictating shoot stem cell initiation and proliferation, and WUS proteins assumes a crucial role in shaping the spatial expression profile of *CLV3* in the stem cell region of the outer SAM layer (Zhou et al., 2018; Han et al., 2020a). In a specific context, the WUS protein initiates the activation of *CLV3* exclusively within the CZ, where HAM1/2 proteins are absent. Conversely, HAM1/2 functions as gatekeepers, maintaining *CLV3* expression at subdued levels within the RZ. This dual action effectively prevents the WUS-dependent activation of *CLV3* and/or repressing *CLV3* transcription (Zhou et al., 2018; Han et al., 2020b; Geng and Zhou, 2021). Moreover, the SPLAYED (SYD) protein, serving as an *SNF2* chromatin remodeling ATPase, robustly stimulates *WUS* transcription. In the *syd* mutant, this vital transcriptional activation of *WUS* diminishes, resulting in reduced meristem size (Kwon et al., 2005). Collectively, these factors assume pivotal roles in the maintenance of shoot stem cell homeostasis, with many of their functions intricately entwined with the auxin signaling pathway.

STIMPY protein expression is induced by cytokinin in the *Arabidopsis thaliana* shoot meristem (Wu et al., 2005; Skylar et al., 2010). The deficiency of STIMPY, as evident in the *stimpy* mutant, leads to decreased expression of *ARR5*, culminating in a smaller SAM and pronounced cellular differentiation. Remarkably, this phenotypic manifestation bears a striking resemblance to that of the *wus* mutant phenotype, underscoring the collaborative influence of STIMPY within the cytokinin signaling pathway to maintain the activity of shoot stem cells. Additionally, pivotal genes such as *KNOTTED1* in maize (Vollbrecht et al., 2000; Zinkgraf et al., 2017) and *STM* in *Arabidopsis thaliana* (Long et al., 1996) are indispensable for the steadfast development of the meristem, with their functions profoundly intertwined with the cytokinin signaling pathway (Vollbrecht et al., 2000). Furthermore, the mutant of the cytokinin synthesis gene *LONELY GUY (LOG)* exhibits phenotypic parallels to the *wus* mutant, further accentuating the profound impact of cytokinin on shoot stem cell activity (Chickarmane et al., 2012). Furthermore, recent research has unveiled the involvement of epigenetic modification in preserving stem cell fate in the SAM (Ma et al., 2019b). WUSCHEL acts via regulation of histone acetylation to control auxin signaling output in stem cells which allows cells to translate a potent and highly dynamic developmental signal into stable cell behavior (Ma et al., 2019b). **Figure 1B** provides a succinct visual representation of the signaling pathways governing plant shoot meristem stem cells.

## 3 Stem cells of the root apical meristem

Roots are indispensable for plants, as they facilitate water and nutrient absorption and promote plant development. In *Arabidopsis thaliana*, roots can be divided into three morphological parts: the meristematic zone (MZ), the transition zone (TZ), and the elongation zone (EZ). The meristematic zone can be subdivided into the distal meristem (DM) and the proximal meristem (PM). Within the MZ, we find the stem cell niche, comprising the quiescent center (QC) at its core, surrounded by various stem cell populations. A longitudinal section of the *Arabidopsis thaliana* root reveals that these surrounding stem cell populations consist of cortex or endodermis initials (CEIs), epidermis or lateral root cap initials (ELRCIs), columella initials (CIs), and stele initials (SI). These regions host complex regulatory networks to maintain QC stability and ensure normal apical meristem development (Lee et al., 2013). The formation of the apical QC and its surrounding stem cell population is essential for establishing the apical meristem (van den Berg et al., 1995; Sarkar et al., 2007) (**Figure 1C**).

As in the case of SAM, root apical meristem formation occurs during embryogenesis with the plant hormone auxin playing a crucial role in initiating the apical meristem (Lau et al., 2008; De Smet, 2010; Brumos et al., 2018; Gomes and Scortecci, 2021). The initiation of embryonic root stem cells is regulated by ARF5, which interacts with its auxin-labile inhibitor IAA12/BODENLOS (BDL) (Rademacher et al., 2012). Additionally, ARF5 promotes auxin transport from the embryo to the hypophysis precursor by positively regulating the expression of the auxin transporter *PIN1*, thus providing signals for initiating root apical stem cells (Benkova et al., 2003; Friml et al., 2004; Weijers et al., 2005; Weijers et al., 2006).

Furthermore, *TMO5* and *TMO7*, the members of the bHLH transcription factor family, are regulated by ARF5 and function downstream of the *ARF5-BDL* auxin signaling pathway, controlling the initiation of root apical stem cells (Schlereth et al., 2010). The *ARF5-BDL*-mediated auxin signaling pathway also regulates the initiation of root stem cells through its downstream genes, such as *OBERON1* (*OBE1*), *OBE2*, *TITANIA1* (*TTA1*), *TTA2*, and *NO transmit TRACT* (*NTT*). Mutations in *OBE1/2* and *TAA1/2* lead to delayed root tip development or even root-less phenomenon, akin to the *mp* mutant phenotype (Saiga et al., 2008; Thomas et al., 2009; Saiga et al., 2012). Significantly, ARF5/MP can directly bind to *NTT* regulatory sequences, promoting its expression during root apical meristem initiation. NTT encodes a putative zinc-finger transcription factor, and its presence is essential for apical meristem formation (Crawford et al., 2015).

To maintain stem cells in the root apical meristem, ensuring the stable distribution of QC cells is essential. This stability and the normal development of roots hinge on the consistent arrangement of QC cells and surrounding stem cells in the root apical meristem. While other plant species often exhibit more complex root structures than *Arabidopsis thaliana*, the straightforward organization of *Arabidopsis* roots has made it an ideal model for plant stem cell research (Timilsina et al., 2019; Wein et al., 2020).

Laser ablation techniques provided insight into the pivotal role of QC in controlling stem cell activity (van den Berg et al., 1997). Following the ablation of QC cells, adjacent columella stem cells (CSCs) cease proliferation and differentiate into starch-containing columella cells. Simultaneously, cortex and endodermis initials (CEIs) neighboring the ablation differentiate into CEI daughter cells. Importantly, stem cell activity is maintained only when cells are in direct contact with QC. RNAi-mediated downregulation of *RETINOBLASTOMARELATED* (*RBR*) activity impedes the differentiation of stem cell progeny, resulting in several layers of undifferentiated cells adjacent to the QC. This experimental evidence highlights the pivotal role of QC-delivered stem cell-promoting signals, which are effective within a certain range of cell diameters and typically diminish in cells not in direct contact with the QC. Therefore, the QC-delivered stem cell-promoting signal represents the essential pathway for stem cell maintenance in *Arabidopsis thaliana* root tips (Dubrovsky and Ivanov, 2021; Matosevich and Efroni, 2021; Strotmann and Stahl, 2021; Zhai et al., 2023).

To ensure the normal growth of plants, it is imperative to uphold the unique characteristics of stem cells in the root apical meristem and SAM during the postembryonic growth phase (Yang et al., 2019; Yu et al., 2019). The *WUSCHEL-RELATED HOMEOBOX5 (WOX5)* gene, serving as a specific marker of the QC within the root apical meristem, plays a crucial role in regulating the equilibrium between cell division within the apical meristem and stem cell differentiation. *WOX5* ensures the persistence of terminal stem cells (Sarkar et al., 2007), as evidenced by the abnormal phenotype observed in *wox5* mutants, characterized by premature differentiation of columella cells. Conversely, the restoration of *WOX5* expression results in delayed columella cell differentiation (Zhang et al., 2015; Zhai et al., 2020).

The maintenance of the QC necessitates the functioning of the *PLT* and *SHR/SCR* pathways. A dearth of gene expression within these two pathways can engender disruptions in QC cell distribution and the untimely cessation of root growth. The *PLT* gene encodes a transcription factor featuring the *AP2* domain, fervently promoting cell division within the root meristem. The loss of *PLT* gene expression can lead to the loss of the radicle, the embryonic root. In concert, *SHR* and *SCR*, members of the GRAS transcription factor family, maintain a regulatory relationship, with SHR being the catalyst for *SCR* transcription. Subsequently, SCR perpetuates QC stability, independent of cell-cell interactions (Sablowski, 2007; Petricka et al., 2012). Additionally, regulating the nuclear localization of *SHORTROOT* (*SHR*) emerges as a pivotal contributor to the maintenance of the root meristem (Sabatini et al., 2003).

An intriguing observation pertains to the existence of a negative feedback regulatory pathway involving *WOX5/ACR4/CLE40* in the root apical meristem. *CLE40*, a polypeptide akin to *CLV3*, inhibits the expression of *WOX5* when excessively secreted by columella cells. This inhibition, in turn, mitigates stem cell proliferation and fosters cell differentiation within the root meristem, providing a critical regulatory mechanism (Stahl et al., 2009). Another significant contributor is Repressor of WUSCHEL1 (ROW1) protein. Its absence precipitates the ectopic expression of *WOX5*, ensuing disruption within the QC region and perturbing cell differentiation (Zhang et al., 2015). Moreover, WOX5 serves as a guardian of the root stem cell niche identity, accomplished through the repression of the differentiation factor *CDF4* or the inhibition of *CYCD1;1* and *CYCD3;3* activity (Forzani et al., 2014; Pi et al., 2015).

Plant hormones, especially auxin and cytokinin, are intricately entwined in the preservation of stem cells in the root apical meristem (Artner and Benkova, 2019; Roychoudhry and Kepinski, 2022). Auxin regulates QC stability and meristem activity through three gene classes: *PLT*, *ARF*, and *PID* (Aida et al., 2004; Friml et al., 2004; Galinha et al., 2007). Among these, *PLT1, PLT2, PLT3*, and *BBM* genes are promoted by auxin concentration gradients mediated by the *PIN* protein. They are also positively regulated by auxin response factors, such as ARF5 and ARF7, contributing significantly to early radicle development and stem cell maintenance (Aida et al., 2004; Galinha et al., 2007). PLT gradient formation is associated with *de novo* organ development. The auxin–PLT network can act as a core module to regulate growth. Meanwhile, auxin-PLTs-ARRs molecular network controls the self-organized patterning of the root (Mahonen et al., 2014; Santuari et al., 2016; Salvi et al., 2020). Recent findings have revealed that auxin stimulates the expressions of tyrosylprotein sulfotransferase (TPST) and several *ROOT GROWTH FACTOR* (*RGF*) genes, thus maintaining the characteristics of root stem cells by inducing a gradient of *PLT* expression (Matsuzaki et al., 2010; Zhou et al., 2010). In contrast, cytokinin fosters cell differentiation, stifling root apical meristem division and expansion (Lee et al., 2013). Moreover, cytokinin has been found to mediate cell differentiation in the root meristem through the regulation of PIN5-mediated auxin intracellular transporter or via IAA-amino synthase *GRETCHEN HAGEN 3.17* (*GH3.17*)-mediated auxin homeostasis (Di Mambro et al., 2019). At the same time, cytokinin can downregulate the expressions of *PIN1* and *PIN4* while positively upregulating the expression of *PIN3* and *PIN7*, thereby orchestrating the size and activity of the root meristem (Ruzicka et al., 2009; Scacchi et al., 2010). Cytokinin activates the expressions of *ARR1* and *ARR2* proteins and subsequently induces the upregulation of *IAA3*, which, in turn, promotes cell differentiation in the root tip transition region (Dello Ioio et al., 2008; Moubayidin et al., 2010). Furthermore, cytokinin and auxin cooperate to maintain the size of the root meristem and ensure root growth through the intermediary action of the *SHY2* protein, a repressor of auxin signaling which negatively regulates the PIN, that is activated by either *ARR1* or *ARR12* (Dello Ioio et al., 2008; Moubayidin et al., 2010). A comprehensive model elucidating the signaling pathways of plant root meristem stem cells is displayed in **Figure 1D**.

## 4 Plant abiotic stresses

The preceding section provided an exhaustive examination of the architecture, initiation processes, and maintenance mechanisms of plant meristem stem cells. In this section, we delved into the kingdom of plant responses to abiotic stresses, with a particular emphasis on elucidating the intricate mechanisms governing how plants perceive and respond to these environmental challenges. Additionally, we explored the intriguing interconnections between the development of plant stem cells and their responses to various abiotic stressors (Zhu, 2016; Gong et al., 2020; Zhang et al., 2020a; Ubogoeva et al., 2021).

We emphasized the plant’s ability to perceive these stressors through specialized molecular pathways, including osmolarity, salinity, temperature, and drought. It is noteworthy that each form of abiotic stress engages unique molecular signaling pathways tailored to its specific attributes. To commence our exploration, we scrutinize the intricate process of how plants sense alterations in osmolarity. Both drought and salinity can induce high osmotic stress in plant cells. In *Arabidopsis thaliana*, the *Reduced Hyperosmolality-Induced [Ca^2+^]_i_ Increase1* (*OSCA1*) gene encoding the hyperosmolarity-gated calcium channel takes on the role of an osmotic pressure sensor. In *osca1* mutants, intracellular Ca^2+^ influx is reduced, leaf transpiration is deficient, and plant root growth is inhibited under high osmotic pressure (Jojoa-Cruz et al., 2018; Liu et al., 2018b; Maity et al., 2019). Similarly, in *Arabidopsis thaliana*, the *MSCS-LIKE 8* (*MSL8*) gene, encoding the membrane tension-gated ion channel, functions as a sensor of pollen membrane tension induced by low osmotic stress. Under low osmotic pressure, *MSL8* expression is upregulated, facilitating ion outflow and safeguarding cells against hypoosmotic stress (Hamilton et al., 2015). These two genes assume pivotal roles in sensing osmotic pressure signals in plants.

Transitioning to the realm of salinity perception, when plants confront high-salt environments, they grapple not only with elevated osmotic stress but also with ion stress induced by Na^+^ ions. Typically, changes in Na^+^ signaling pathways coincide with Ca^2+^ signaling pathways. In *Arabidopsis thaliana*, the *Monocation-Induced [Ca^2+^]_i_ Increases1* (*MOCA1*) gene encodes a glucuronodyl transferase responsible for appending a negatively charged glucuronic acid (GlcA) group to inositol phosphorylated ceramide (IPC), resulting in the formation of glycosyl inositol phosphorylated ceramide (GIPC) sphingolipids that bind Na^+^ cations (Jiang et al., 2019). Research has revealed that MOCA1-dependent GIPC serves as a sensor for fluctuations in environmental Na^+^ levels, with this process entailing Ca^2+^ transporters. In *Arabidopsis thaliana*, two highly bona fide Ca^2+^ permeable transporters, ANNEXIN1 (AtANN1) and AtANN4, come into play (Laohavisit et al., 2013; Ma et al., 2019a). AtANN1 is indispensable for facilitating salt-activated Ca^2+^ inflow into the plasma membrane of root epidermal cells, while AtANN4, in conjunction with its interacting proteins Salt Overly Sensitive2 (SOS2) and SOS3-Like Calcium Binding Protein8 (SCaBP8) (also known as CBL10), regulates Ca^2+^ signaling changes induced by salt stress, facilitated through a phosphorylation-dependent negative feedback loop (Ma et al., 2019a). Salt stress not only perturbs the plasma membrane but also disrupts cell wall integrity through various mechanisms. In *Arabidopsis thaliana*, the LRX-RALF-FER module, including the Cell Wall Leucine-Rich Repeat Extensins3/4/5 (LRX3/4/5), Rapid Alkalinization Factor22/23 (RALF22/23), and Receptor-Like Kinase FERONIA (FER) proteins, emerges as a pivotal component in sensing salt stress and regulating salt tolerance (Feng et al., 2018; Zhao et al., 2018a).

Temperature variations, encompassing both heat and cold stress, can modulate the fluidity of the plasma membrane. Cold stress perception predominantly centers on Ca^2+^ channels in the plasma membrane. In rice, mutations in Cyclic Nucleotide-Gated Channel (CNGC) ion channel proteins, specifically OsCNGC9, OsCNGC14, and OsCNGC16, alter temperature stress-induced Ca^2+^ signaling and reduce tolerance to temperature stress (Ma et al., 2015; Cui et al., 2020; Liu et al., 2021; Wang et al., 2021). Notably, recent research has unveiled the *Chilling tolerance divergence 1* (*COLD1*) gene as a potential low-temperature sensor. *COLD1*, in concert with *Rice G-protein-α subunit 1* (*RGA1*), triggers a cold stress-induced increase in cytoplasmic Ca^2+^ levels (Ma et al., 2015). These findings underscore the critical role of Ca^2+^ signaling in temperature perception. Intriguingly, in *Arabidopsis thaliana*, diminished Ca^2+^ influx induced by cold stress is observed in *atann1* mutants, resulting in reduced cold tolerance. Remarkably, *AtANN1*, involved in temperature perception, is also implicated in salinity perception in *Arabidopsis thaliana*, hinting at potential shared signaling pathways for temperature and salinity perception (Laohavisit et al., 2013). In addition, nuclear and cytoplasmic proteins, such as the histone variant H2A.Z and the photoreceptor phytochrome B (phyB), may contribute to temperature sensing (Kumar and Wigge, 2010; Jung et al., 2016; Legris et al., 2016). H2A.Z has been demonstrated to regulate gene transcription in a temperature-dependent manner (Kumar and Wigge, 2010), while phyB responds to changes in ambient temperature by altering its active state (Jung et al., 2016; Legris et al., 2016; Fujii et al., 2017). These findings suggest that photoreceptors like phyB may assume crucial roles in sensing extreme temperature fluctuations. In *Arabidopsis thaliana*, heat stress perception is mediated by the transcriptional suppressor *Early Flowering 3* (*ELF3*). *ELF3* is involved in regulating the expressions of numerous genes associated with growth and development. As temperatures rise, *ELF3* gradually loses its inhibitory effect on downstream target genes, resulting in their activation (Jung et al., 2020). Notably, both heat and cold stress can affect various physiological and biochemical properties of plants. Among these, protein denaturation, a phenomenon unique to heat stress, triggers a specific response mediated by heat shock proteins (HSPs). These HSPs, typically inhibiting heat stress transcription factors (HSF) at normal temperatures, relinquish their hold on HSF as temperature rises, owing to the accumulation of denatured proteins. This event initiates the heat stress response (Scharf et al., 2012).

**Figure 2:**
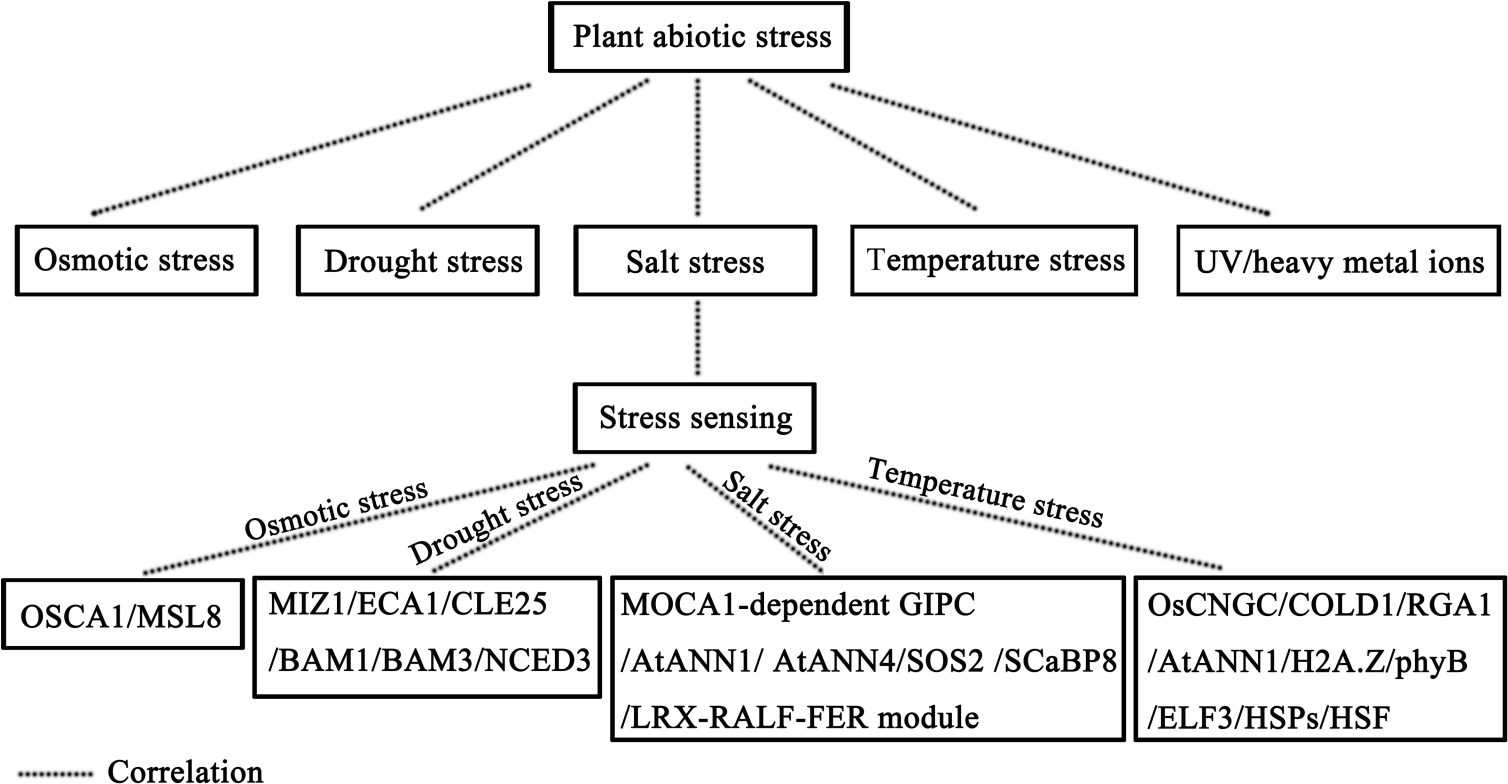
Plant abiotic stress models.

In summation, plants have exhibited a remarkable capacity to perceive abiotic stresses, such as osmotic stress, salinity stress, and temperature stress, through different signaling pathways. These perceptive processes span from ion channels at the plasma membrane, including Na^+^ and Ca^2+^ channels, to components at the cell surface, such as cell walls, and extend to intracellular compartments, encompassing the cytoplasm and nucleus. The profound hydrophilic nature of plants, manifested through their preference for habitats with high water potential, underscores their ability to sense soil moisture levels effectively. The timely activation of stress response mechanisms is essential for plant survival under drought conditions. Recent studies have indicated that Ca^2+^ signaling is also involved in the root hydrophilic response (Shkolnik et al., 2018). *miz1* mutants, characterized by diminished hydrophilicity, exhibit weakened phloem Ca^2+^ signals. In contrast, mutations affecting the *ER-localized type 2A Ca^2+^-ATPase* (*ECA1*), which interacts with Mizu-Kussey 1 (MIZ1) and is inhibited by MIZ1, result in elevated Ca^2+^ levels and heightened hydrophilicity (Shkolnik et al., 2018). Therefore, *MIZ1* and *ECA1* emerge as key determinants influencing root hydrophilicity. However, the precise mechanisms underlying the perception of water potential and drought conditions warrant further experimental validation. As the root system senses water deficit in the soil, it can transmit this signal to aerial parts of the plant. While the exact mechanism governing this perception remains the subjects of ongoing investigation, a wealth of evidence implicates abscisic acid (ABA), H^+^ (pH), Ca^2+^, ROS, NO, lipids, small peptides, RNA molecules, and physical signals participating in this process. Of particular note, the small peptide *CLAVATA3*/*endosperm surrounding region-related 25* (*CLE25*) has been observed to intensify in response to plant dehydration. Upon interaction with receptors Barely Any Meristem 1 (BAM1) and BAM3, *CLE25* stimulates the expression of the *Nine-Cis-Epoxycarotenoid Dioxygenase 3* (*NCED3*) gene, thereby promoting ABA biosynthesis and enhancing drought tolerance (Takahashi et al., 2018). While substantial progress has been made in comprehending some perception mechanisms, numerous facets of these processes merit further dedicated investigation. **Figure 2** provides a concise visual representation of plant responses to abiotic stresses.

## 5 Mechanisms governing plant stem cell responses to abiotic stresses

In the context of their sessile nature, plants confront environmental challenges by deploying intricate survival mechanisms. Central to these adaptive strategies are meristem cells, the linchpin of plant development governing roots, shoots, leaves, and flowers (Wang et al., 2020). However, meristem cells are acutely sensitive to abiotic stresses, including temperature fluctuations (both cold and heat), salinity, drought, ultraviolet radiation, and irradiation. These factors can inflict DNA damage and disrupt the normal division of stem cells, precipitating either stem cell mortality or aberrant plant development. Nevertheless, the plant kingdom has honed an assortment of mechanisms to ensure the persistence of stem cell activity, equipping them to survive amidst ever-changing environments. Presented below is a comprehensive elucidation of these mechanisms.

In the face of prolonged or severe stress conditions, apical stem cells manifest distinctive. Initially, the activity of root apical stem cells may dwindle or, in extreme cases, succumb to apoptosis. Concurrently, this milieu incites the division of QC cells. Etiological agents such as ultraviolet radiation, diverse forms of irradiation, and specific pharmaceutical agents, including bleomycin and zeocin, serve as instigators of DNA damage and consequent apoptotic responses in apical stem cells (Fulcher and Sablowski, 2009; Furukawa et al., 2010; Heyman et al., 2013; Canher et al., 2020). Perturbations in temperature, especially cold stress, may similarly usher in the demise of root stem cells. Nevertheless, in most cases, plants sacrifice selects columella stem cell progeny to ensure the survival of pivotal stem cells (Hong et al., 2017). Prolonged exposure to high-intensity radiation elicits QC cell division, with QC cells exhibiting greater resilience to DNA damage when juxtaposed with stem cells. QC cell demise typically materializes only under severe stress circumstances (Fulcher and Sablowski, 2009; Furukawa et al., 2010). Simultaneously, in instances of severe damage, such as the removal of the root cap (Ivanov et al., 2011; Bystrova et al., 2015), exposure to chilling stress (Hong et al., 2017), significant alterations in water osmotic potential (Mira et al., 2020), exposure to heavy metal ions (Kozhevnikova et al., 2007), and heat stress (Heyman et al., 2013; Heyman et al., 2016), the activation of QC cell division is observed, facilitating the restoration of root growth.

Importantly, the NAC family transcription factor *SUPPRESSOR OF GAMMA RESPONSE1* (*SOG1*) assumes a central role in orchestrating plant stem cell responses to high-intensity UVB radiation, X-rays, and radiological agents (Fulcher and Sablowski, 2009; Furukawa et al., 2010; Ogita et al., 2018; Ryu et al., 2019). Root tip responses to radiation and aluminum stress also entail the cooperative engagement of two cell cycle checkpoint kinases, ATAXIA TELANGIECTASIA MUTATED (ATM) and ATM AND RAD3-RELATED (ATR) and aluminum stress (Rounds and Larsen, 2008; Sjogren et al., 2015).

Simultaneously, the transcription factor *ETHYLENE RESPONSE FACTOR 115* (*ERF115*) assumes prominence, as it emerges as a lynchpin in facilitating plant recovery and regeneration amidst stressful conditions. Under standard conditions, *ERF115* remains confined to QC cells, serving as a rate-determining regulator of QC division (Heyman et al., 2013). Upon exposure to stressors such as elevated temperatures or physical injury, *ERF115* can express not only in QC cells, but also in adjoining areas of cell demise, and the inner cortex. This activation instigates a cascade effect, prompting stem cells to engage in restorative cell division (Heyman et al., 2013; Heyman et al., 2016; Zhou et al., 2019b; Canher et al., 2020). *ERF109*, a homologous gene of *ERF115*, also features prominently, as it experiences rapid induction following programmed cell death, thereby stimulating the expression of *ERF115* in the proximity of dead cells and fostering the meristem regeneration (Heyman et al., 2016; Zhou et al., 2019b). Additionally, *ERF115* forms heterodimers with *PHYTOCHROME A SIGNAL TRANSDUCTION1* (PAT1), hereby expediting restorative cell division subsequent to root tip excision (Heyman et al., 2016).

The pivotal distribution of reactive oxygen species (ROS) profoundly influences the adaptative responses of stem cell niches to stressful conditions. ROS, metabolic by-products of aerobic metabolism, accumulate under stressful conditions, culminating in DNA damage, protein oxidation, and lipid peroxidation (Gill and Tuteja, 2010; Huang et al., 2019). Stressors, such as heat, cold, drought, heavy metals, and pathogens, rapidly perturb the redox homeostasis, resulting in pronounced ROS accumulation in plant tissues. This perturbation, in severe cases, may culminate in the senescence of plant organs (Lee et al., 2012; Kawarazaki et al., 2013; Kim and Hwang, 2014; Zhao et al., 2018b). A typical example is the flood-induced hypoxic response of apical stem cells. Under hypoxic conditions, maintaining low levels of ROS is essential for safeguarding apical meristem persistence (Sasidharan et al., 2018). In the apical meristem, both hypoxia-induced ROS and nitric oxide (NO) accumulation elicit QC cell division and meristem cell death (Mira et al., 2016). *Prohibitin3* (*PHB3*), in this context, can restrict the spatial expression of ethylene response factor (ERF) transcription factors, ERF115, ERF114, and ERF109. These ERF factors, in turn, mediate ROS signaling in a PLT-independent manner to regulate the maintenance of stem cell niches and root growth through phytosulfokine (PSK) peptide hormones (Kong et al., 2018). Recent investigations have underscored the pivotal role of the *Arabidopsis SYNTAXIN OF PLANTS81(AtSYP81)* gene in root development, where it regulates peroxisome-mediated ROS production in the root tip. In *atsyp81* mutants, a noticeable reduction in ROS levels is observed in the root tips, resulting in a significant decrease in apical meristem activity and the disruption of the apical stem cell niche’s identity. The supplementation of ROS donors in *atsyp81* mutants effectively rectifies the impaired stem cell viability in the apical meristem. These experimental findings indicate the role of AtSYP81 in regulating root meristem activity and preserving the identity of the apical stem cell niche through the control of peroxisomes and peroxidase-mediated ROS homeostasis (Wang et al., 2023). In *Arabidopsis thaliana*, ROS signaling is critical for meristem development and the maintenance of stem cell identity (Ubogoeva et al., 2021). A variety of stressors can disrupt the balance of ROS in plants, leading to damage or even the senescence of plant organs. Consequently, the maintenance of a balanced ROS level assumes paramount importance for the survival of plant stem cells.

Soil salinity affects plant development, invariably diminishing crop yield. Recent scientific inquiries have shed light on the response to NaCl concentrations surpassing a specified threshold in the root tip of *Arabidopsis thaliana*. This incites the activation of local and systemic Ca^2+^ signaling pathways, which culminate in the engagement of the Salt Overly Sensitive (SOS) pathway. The SOS pathway is a conserved and important regulatory mechanism for Na^+^ exclusion and for alleviating long-distance transport of toxic Na^+^ (Shi et al., 2002; El Mahi et al., 2019). The latter comprises an ensemble of Ca^2+^ sensors, kinases, and Na^+^/H^+^ exchange modules that are ubiquitously distributed throughout the root. In concert with this, salt stress occasions a significant upregulation of *GSO1* expression, especially in the endodermis and meristem (Chen et al., 2023). The upregulated GSO1 expression in the apical meristem, in particular, serves as a specialized conduit for intracellular Na^+^ detoxification *via* the GSO1-SOS2-SOS1 module. Activation of the SOS2-SOS1 module allows root growth by protecting the meristem to be maintained in adverse environments (Chen et al., 2023). This multifaceted receptor-like kinase *GSO1*-mediated mechanism is pivotal in preserving intracellular ion homeostasis and, by extension, normal root development, thereby enabling roots to sustain growth in adverse environments (Chen et al., 2023). Concurrently, it is imperative to recognize that the resilience and adaptability of plant apical meristem cells to salt stress are inextricably linked with microRNAs, the redox state, ROS, NO, and plant hormones such as auxin and cytokinin (Yang and Lee, 2023). Recent research has found that a prion-like domain (PrD) in the key shoot meristem regulator STM can stimulate it to form nuclear condensates which are required for maintaining the shoot meristem. The formation of STM condensates is enhanced upon salt stress, which allows enhanced salt tolerance and increased shoot branching (Cao et al., 2023). Salt-dependent reduction of miR165 and 166 causes a rapid increase in PHABULOSA (PHB) expression and production of the root meristem prodifferentiation hormone cytokinin (Scintu et al., 2023). A comprehensive metabolome and transcriptome analysis of three CK signal-deficient Arabidopsis *ahp2,3,5* and *arr1,10,12* mutants treated with salt stress showed that CK signaling induced reprogramming of gene-metabolic networks associated with Arabidopsis response to salinity (Abdelrahman et al., 2021). This intricate nexus highlights the correlation between salt stress response and signaling cascades that govern stem cell homeostasis in the plant meristem (Yang and Lee, 2023).

Temperature fluctuations exert profound control over plant development by modulating the behavior and attributes of stem cells. The programmed cell death of columella stem cell daughters (CSCDs) reestablishes the auxin maximum in the QC and preserves the functionality of the stem cell niche during chilling stress. This regulatory mechanism substantially enhances the survival prospects of plant roots when confronted with chilling stress. Furthermore, upon the restoration of normal ambient temperatures, the plant’s root system can recommence its growth phase (Hong et al., 2017).

In higher plants, the SAM perpetuates a division-centric agenda that culminates in the development of aerial plant organs. However, water assumes paramount importance in maintaining normal plant development, and drought stress can impede the proliferation of plant stem cells. In *Arabidopsis thaliana*, the *STM* gene emerges as a critical regulator in the SAM, enhancing plant tolerance to drought. In the shoot tips of *Arabidopsis thaliana*, the R2R3-type MYB96 transcription factor, inducible by ABA, plays a pivotal role in adapting to drought stress by regulating the transcriptional accumulation of *STM*. The overexpression of *MYB96* substantially upregulates the expression of *STM*, thereby engendering enhanced drought tolerance. Conversely, *myb96* mutants exhibit a marked reduction in STM expression, concomitant with diminished drought tolerance. These observations underscore the pivotal role played by the *MYB96-STM* axis in bolstering plant tolerance to drought stress (Lee et al., 2016).

The SAM in plants navigates a complex regulatory network that balances proliferation and differentiation processes. Cell differentiation in the developing shoot is intrinsically tied to the intracellular environment and the role of plastids. *Arabidopsis thaliana* mutants exhibiting chronic osmotic stress (*msl2 msl3*) manifest significantly enlarged and morphologically aberrant plastids in the SAM, concomitant with the formation of callus in large quantities at the shoot tips. This induction of callus formation is underpinned by a nexus of interconnected mechanisms involving cytokinin receptor AHK2, cytokinin signaling inhibitors ARR7 and ARR15, and the stem cell identity gene *WUSCHEL*. Furthermore, the process of callus induction precipitated by plastids under osmotic stress necessitates heightened levels of ROS and ABA biosynthesis in plastids. This intriguing interplay indicates the role of osmotic stress in activating plastid-mediated mechanisms that promote SAM development, implicating the cytokinin signaling pathway in this process (Wilson et al., 2016).

Plant hormones, especially auxin and cytokinin, wield substantial influence over the development of plant meristem cells. These phytohormones, in turn, assume varying roles in the context of stem cell responses to abiotic stresses. Auxin is essential for plants to respond to stress (Jing et al., 2023). Notably, with its maximum concentration dictating QC identity and preserving stem cell niche integrity, emerges as a critical factor in stress adaptation (Jiang and Feldman, 2005). For instance, *TRYPTOPHAN AMINOTRANSFERASE OF ARABIDOPSIS 1* (*TAA1*) in *Arabidopsis thaliana* can promote auxin accumulation in the root tips under aluminum stress (Yang et al., 2014). *TAA/TAR*-mediated auxin biosynthesis emerges as indispensable in the root meristem’s response to ethylene stress (Brumos et al., 2018). Following root damage or removal in *Arabidopsis thaliana*, auxin biosynthesis in the root tips accelerates significantly, expediting the recovery of root growth (Matosevich et al., 2020). The molecular marker DR5, reflective of auxin activity in the root, exhibits decreased activity in the QC under low potassium (K^+^) conditions (Zhang et al., 2020b). Auxin plays an important role in a “sacrifice-for-survival” mechanism in which cold-damaged CSCs are sacrificed in order for the root growth. Low-temperature stress leads to decreased DR5 activity in the QC, which in turn precipitates CSC cleavage (Hong et al., 2017). In addition, auxin has demonstrated a protective role, safeguarding stem cells from Zeocin-induced cell death (Hong et al., 2017). The homeodomain TF gene, HB33 was identified as a positive regulator of ABA response which was repressed by auxin response factor 2 (ARF2). The *arf2* mutant showed enhanced ABA sensitivity (Wang et al., 2011). ARF2-HB33 module may link the ABA and auxin signaling pathways to response the drought stress (Wang et al., 2011). Overexpression of auxin biosynthesis genes leads to increased salt tolerance (Dunlap and Binzel, 1996; Kim et al., 2013; Ke et al., 2015). The expression of auxin biosynthesis gene *YUC4* is significantly increased in response to NaCl treatment in Arabidopsis (Cackett et al., 2022). The *tir1/afb2/afb3* mutants, which was defective in multiple TIR/Auxin-Signaling F-box (AFB) receptors, display hypersensitivity to NaCl treatment in the RAM. The slowing of root growth caused by salt stress might be an adaptive mechanism for plants surviving (Iglesias et al., 2010; Yu et al., 2020).

In addition to auxin, the plant stem cell niche is profoundly influenced by other phytohormones, such as cytokinin, ethylene, jasmonic acid (JA), and brassinosteroid (BR). Recent investigations have revealed cytokinin’s integral role in fortifying plant responses to temperature stress, drought stress, and salt stress (Li et al., 2021b). This phytohormone’s signaling pathway encompasses *C*YTOKININ RESPONSE FACTOR (CRF), ARABIDOPSIS HISTIDINE CONTAINING PHOSPHOTRANSMITTER (AHP), cytokinin receptor (AHK), and ARABIDOPSIS RESPONSE REGULATOR (ARR) proteins, collectively steering plants towards tolerance and adaptability of plants under these adverse conditions (Jeon et al., 2010; Mason et al., 2010; Kang et al., 2012; Chen and Yang, 2013; Jeon and Kim, 2013; Nishiyama et al., 2013; Jeon et al., 2016; Joshi et al., 2016; Nguyen et al., 2016; Cortleven et al., 2019; Song et al., 2019; Zhou et al., 2019a; Hyoung et al., 2020; Li et al., 2021a; Li et al., 2021b). Similarly, ethylene accumulation in the root tips in response to soil compaction or flooding underscores ethylene’s pivotal role in aiding meristem adaptation to stress (Hartman et al., 2019; Pandey et al., 2021). Ethylene signaling, in this context, is integral to the scavenging of the plant globin1 (PGB1), a process essential for meristem adaptation to flood-induced hypoxia (Hartman et al., 2019). Recent studies have shown that JA plays a key role in stem cell niche regeneration (Zhou et al., 2019b). In instances where the root tip is cut off or infected by pathogens, JA and auxin synergistically activate the *SCR-SHR-RBR* pathway, facilitating restorative cell division and the resumption of root tip growth.

**Figure 3:**
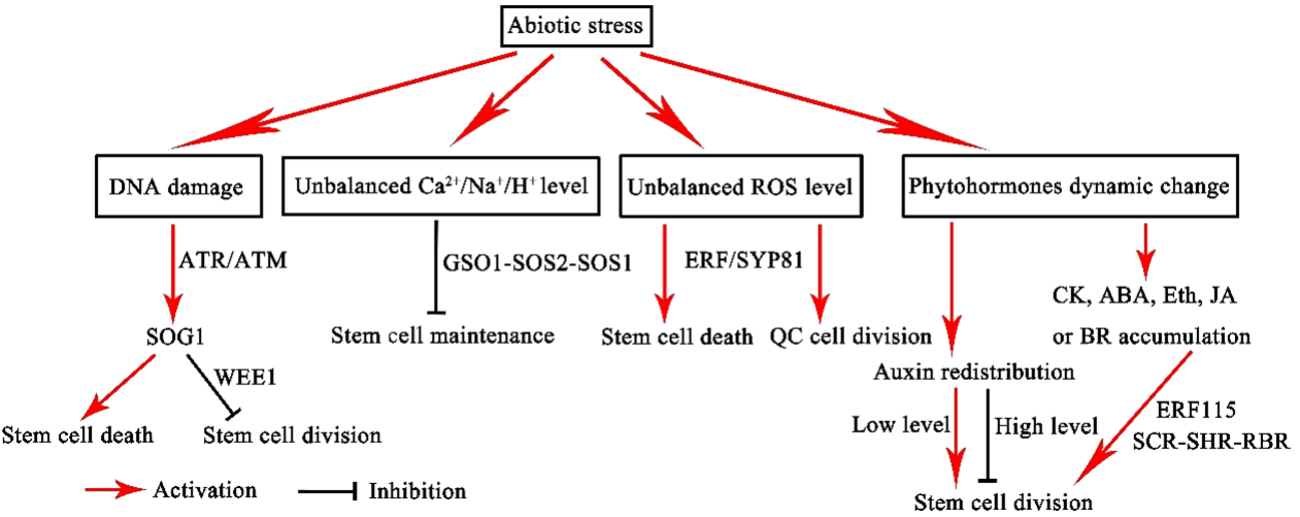
Model of the stem cell niche response to abiotic stress. ROS, reactive oxygen species; CK, cytokinin; ABA, abscisic acid; Eth, ethylene; JA, jasmonic acid; BR, brassinosteroid.

Furthermore, BR recruitment of the BRI1-EMS-SUPPRESSOR 1 (BES1) -BRASSINOSTEROIDS AT VASCULAR AND ORGANIZING CENTER (BRAVO) -ERF115 signaling module assumes a critical role in governing QC cell division and sustaining the characteristics of the apical meristem (Vilarrasa-Blasi et al., 2014). The dynamic involvement of BR signaling in plant responses to temperature, salt, and drought stresses further underscores its multifaceted influence on plant development (Planas-Riverola et al., 2019). The intricate web of responses orchestrated by plant stem cells in the face of abiotic stresses is displayed in **Figure 3**.

## 6 Conclusions and perspectives

The initiation and maintenance of plant meristem cells represent vital processes governing plant growth and development. Advancements in technologies such as single-cell sequencing have yielded an expanding body of experimental evidence supporting the crucial role of plant stem cells, extending their implications to plant regeneration and crop yield improvement (Liu et al., 2023). These developments have contributed to a more comprehensive grasp of the initiation, maintenance, signal transduction, and other processes of plant stem cells. In the backdrop of ongoing global environmental shifts, characterized by phenomena such as drought stress, temperature fluctuations, salt stress, and exposure to heavy metal ions, plants have undergone intricate adaptations in regulatory mechanisms to safeguard their reproductive integrity. As the core components of plant development, plant stem cells play a pivotal role in enabling plants to bolster their resilience against environmental stresses. This review has furnished a comprehensive exploration of the regulatory mechanisms governing the initiation and maintenance of plant stem cells, including the root apical meristem and the shoot apical meristem. Additionally, we have summarized the response mechanisms that plants employ to counter abiotic stresses, with a particular emphasis on the processes of perception in these stresses. Furthermore, we have outlined recent insights into the mechanisms by which plant stem cells fortify tolerance and adaptability under challenging conditions. However, it is imperative to acknowledge that the study of plant responses to abiotic stresses remains an ongoing pursuit, rife with numerous outstanding questions. These inquiries span areas such as the identification of receptors that sense stress signals, the unraveling of the mechanism governing apical meristem of plant responses to stress, and the exploration of unresolved aspects of abiotic stresses that have surfaced in recent literature (Verslues et al., 2023). Solutions to these important scientific questions will undoubtedly proceed in tandem with the relentless advancement of scientific knowledge.

## Author contributions

HKZ: Writing – original draft, Writing – review & editing. YM: Writing – review & editing. HZ: Writing – original draft, Writing – review & editing. CY: Writing – original draft, Writing – review & editing.
